# Radiomics and Machine Learning Can Differentiate Transient Osteoporosis from Avascular Necrosis of the Hip

**DOI:** 10.3390/diagnostics11091686

**Published:** 2021-09-15

**Authors:** Michail E. Klontzas, Georgios C. Manikis, Katerina Nikiforaki, Evangelia E. Vassalou, Konstantinos Spanakis, Ioannis Stathis, George A. Kakkos, Nikolas Matthaiou, Aristeidis H. Zibis, Kostas Marias, Apostolos H. Karantanas

**Affiliations:** 1 Department of Medical Imaging, University Hospital of Heraklion, 71110 Heraklion, Crete, Greece; miklontzas@ics.forth.gr (M.E.K.); vassalou.e@hotmail.com (E.E.V.); vispan@windowslive.com (K.S.); stathisjohn@yahoo.gr (I.S.); drkakkosgeorge@gmail.com (G.A.K.); nikolas_mat@hotmail.com (N.M.); 2 Computational BioMedicine Laboratory, Institute of Computer Science, Foundation for Research and Technology (FORTH), 70013 Heraklion, Crete, Greece; gmanikis@gmail.com (G.C.M.); nikiforakik@gmail.com (K.N.); kmarias@hmu.gr (K.M.); 3 Advanced Hybrid Imaging Systems, Institute of Computer Science, Foundation for Research and Technology (FORTH), 70013 Heraklion, Crete, Greece; 4 Department of Radiology, School of Medicine, University of Crete, Voutes Campus, 71003 Heraklion, Crete, Greece; 5 Department of Anatomy, Medical School, University of Thessaly, 41334 Larissa, Greece; ahzibis@gmail.com; 6 Department of Electrical & Computer Engineering, Hellenic Mediterranean University, 71004 Heraklion, Crete, Greece

**Keywords:** hip, avascular necrosis of bone, osteoporosis, machine learning, artificial intelligence, transient osteoporosis, radiomics, XGboost

## Abstract

Differentiation between transient osteoporosis (TOH) and avascular necrosis (AVN) of the hip is a longstanding challenge in musculoskeletal radiology. The purpose of this study was to utilize MRI-based radiomics and machine learning (ML) for accurate differentiation between the two entities. A total of 109 hips with TOH and 104 hips with AVN were retrospectively included. Femoral heads and necks with segmented radiomics features were extracted. Three ML classifiers (XGboost, CatBoost and SVM) using 38 relevant radiomics features were trained on 70% and validated on 30% of the dataset. ML performance was compared to two musculoskeletal radiologists, a general radiologist and two radiology residents. XGboost achieved the best performance with an area under the curve (AUC) of 93.7% (95% CI from 87.7 to 99.8%) among ML models. MSK radiologists achieved an AUC of 90.6% (95% CI from 86.7% to 94.5%) and 88.3% (95% CI from 84% to 92.7%), respectively, similar to residents. The general radiologist achieved an AUC of 84.5% (95% CI from 80% to 89%), significantly lower than of XGboost (*p* = 0.017). In conclusion, radiomics-based ML achieved a performance similar to MSK radiologists and significantly higher compared to general radiologists in differentiating between TOH and AVN.

## 1. Introduction

Transient osteoporosis (TOH) and avascular necrosis (AVN) of the hip are two disease entities that have caused great confusion in orthopedic and radiologic literature. Both entities affect the bone marrow of the proximal femur, with TOH causing extensive bone marrow edema (BME) [1,2] and AVN causing necrosis which can manifest with a variety of findings, including BME appearing at later stages of the disease [3,4]. Both diseases can be accompanied by subchondral fractures of different types and can lead to articular collapse if left untreated [2,4,5]. Accurate differentiation between them is greatly dependent on MRI and is of utmost importance since it can either lead to the adoption of conservative treatment for TOH or surgical treatment for AVN. Inaccurate diagnosis may, therefore, have a great impact on the treatment planning since erroneous diagnosis of AVN over TOH can lead to unnecessary surgery.

Confusion has been caused by early publications which suggested a common pathophysiology of the two entities mainly because both can be associated upon MRI with BME, subchondral fractures and articular collapse in advanced disease. Based on these, AVN was thought to be a progression of TOH, causing great confusion for radiologists and orthopedic surgeons [6,7,8]. Nonetheless, current data indicate no pathophysiological similarity between the two diseases, since large cohort results have shown no progression of TOH to AVN [2,5,9], and no microscopic similarities between them [10]. It is also now clear that subchondral fractures in TOH have a completely different morphology compared to the “band-like” and “crescent” sign of AVN. Importantly, TOH has a benign course with complete recovery with only weightbearing protection and painkillers, whereas AVN is not self-limited and requires surgical treatment [2,5,6]. Despite the growing evidence that TOH and AVN should be considered as different disorders, confusion between the two still exists [11,12] and their diagnosis still requires a combination of clinical and imaging features and significant experience in musculoskeletal (MSK) imaging.

Radiomics has emerged as a method for quantitative high-precision image analysis based on high-throughput feature extraction, coupled with advanced machine learning algorithms. Image features invisible to the human eye are extracted and analyzed for comprehensive appraisal of disease states and the identification of data patterns that allow accurate disease diagnosis and monitoring [13,14]. Radiomics attempts to overcome the subjectivity and variability related to image interpretation from radiologists by utilizing multidimensional objective data mathematically derived from images and employing artificial intelligence to analyze the data in an intuitive manner [15]. Radiomics has found limited application in MSK disorders such as the prediction of femoral osteoporosis [16] and the differentiation between low- and high-grade chondrosarcomas [17]. It has also been found to be more accurate than radiologists in distinguishing soft-tissue lipomas from liposarcomas [18] and differentiating between types of sacral tumors [19]. The suitability of radiomics in analyzing bone marrow lesions has been also demonstrated recently by achieving better accuracy than inexperienced radiologists in differentiating bone islands from osteoblastic metastases [20].

Herein, we present the development of radiomics-based machine learning algorithms that aim to differentiate between TOH and AVN. The aim of our study was to extract radiomics features from MR images of patients with both diseases and develop three machine learning models to differentiate between them. The presented machine learning modeling process was based on multivendor images, thus increasing its applicability to MRI examinations from any center. Finally, the performance of developed algorithms was compared to radiologists and radiology residents at different levels of training in an attempt to demonstrate the value of the developed classifiers in the diagnosis of hip bone marrow disease.

## 2. Materials and Methods

### 2.1. Patients

**Figure 1:**
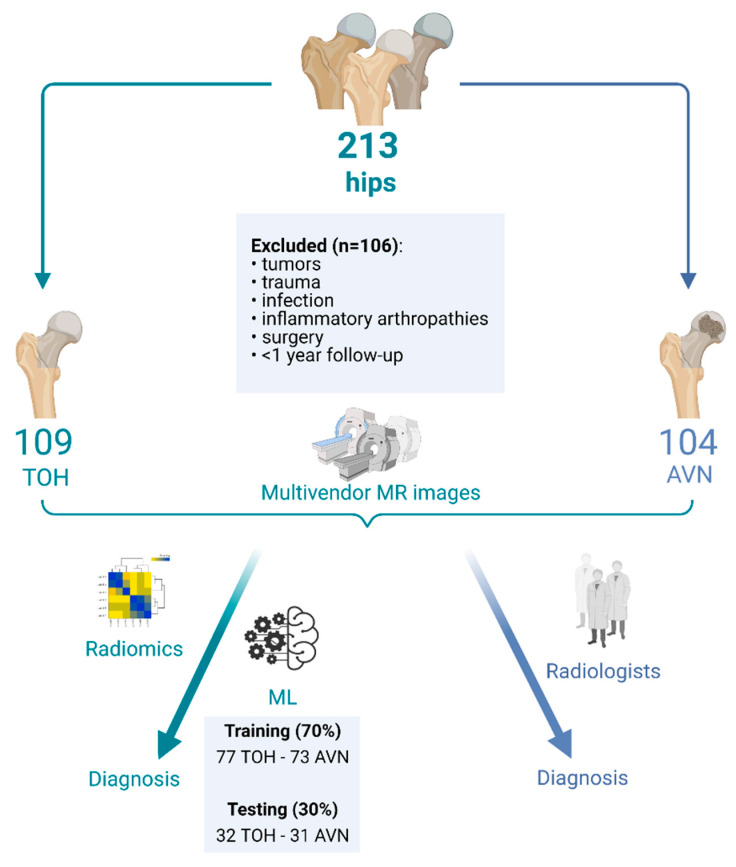
Flow diagram describing the formation of study groups for radiomics analysis and machine learning model development. TOH: Transient Osteoporosis of the Hip; AVN: Avascular Necrosis; ML: Machine Learning (created with BioRender.com, date last accessed: 15 September 2021).

A total of 213 hips were retrospectively included in this study. The dataset comprised 109 consecutive hips with TOH (107 patients) and 104 consecutive hips with AVN (67 patients), referred to the bone marrow imaging specialty referral clinic of a university hospital between July 2014 and March 2020. Patients (n = 106) with tumors, prior trauma, infection, inflammatory arthropathies, follow-up less than 1 year or surgery on the hip of interest were excluded from the study (Figure 1). The study was performed according to the Declaration of Helsinki, all patients have provided informed consent to undergo the examination and the study has received institutional review board approval (Ref. No. 360/08/29-04-2020).

### 2.2. MR Imaging and Ground Truth Diagnosis

For the purposes of initial diagnosis and differentiation between TOH and AVN, MRI findings were evaluated in combination with clinical data including (a) potential risk factors predisposing to AVN and (b) a history of acute or insidious onset of pain extending to the groin and/or thigh indicating TOH compared to AVN, respectively. All TOH patients were followed up for ≥1 year to document spontaneous resolution of symptoms solely with conservative measures, as per routine clinical practice. MRI examinations included in this study were performed in a variety of centers across the country utilizing 1.5 or 3T MRI machines of multiple vendors. A minimum of (a) coronal T1-w, (b) coronal short tau inversion recovery (STIR) sequences, (c) axial fat suppressed PD/T2-w and (d) a high-resolution 3D gradient echo sequence of the affected hip were assessed for each patient. The aforementioned sequences are part of the routine hip protocol in our institution, suitable to evaluate the whole range of hip pathology. In cases of TOH and AVN, only 2D sequences were necessary for the diagnosis. In case the MRI protocol of the initial examination was deemed incomplete, imaging was repeated in-house with a 1.5 T MR scanner (Vision/Sonata, Siemens, Erlangen). All data were evaluated by a senior academic radiologist with 35 years of experience in bone marrow imaging, who evaluated clinical data in conjunction with imaging data. Ground truth diagnosis was made by assessing all available MRI sequences, clinical data at presentation and follow-up, in consensus with the referring orthopedic surgeon and the diagnosis was recorded. The MRI diagnosis of TOH was based on the presence of bone marrow edema, with or without irregular low signal intensity linear structures, deeply located in the subchondral bone [1,2,5]. The presence of a “sparing” sign, joint effusion, synovitis and periarticular soft tissue edema were supportive of the diagnosis (5). The MRI diagnosis of AVN was based primarily on the presence of the “band-like” sign demonstrated with low signal intensity on T1-w MR images and high signal intensity, also known as “single line” sign, on fluid sensitive sequences [1,4,21]. The “double line” sign, originally described in non-fat-suppressed T2-w MR images, is no longer used as it represents a chemical shift artifact [21]. The presence of subchondral fracture was demonstrated with high signal intensity on fluid-sensitive sequences and BME, suggesting an advanced stage of the disease [21]. No alternative method for ground truth establishment exists in clinical practice. For the purposes of radiomics analysis and machine learning model development, mid-coronal STIR MR images through the femoral head and neck were utilized. STIR images suffice for the diagnosis of both AVN and TOH in everyday practice, since fluid-sensitive sequences represent the gold standard for the study of bone marrow edema.

Images used for machine learning model development were also evaluated by radiologists at various levels of training, with and without a special interest in MSK radiology, to compare the ability of a variety of readers to differentiate between the two disorders. In order to capture the whole spectrum of reader experience, images used for model development were also independently reviewed by two fellowship-trained MSK radiologists (E.E.V. and K.S. with 7 and 5 years of MSK experience, respectively), a 4th (I.S.) and a 5th (G.A.K.) year radiology resident with a special interest in MSK radiology and a general radiologist (N.M.). All readers were presented with the same images (randomly shuffled) and were blinded to the ground truth and the performance of machine learning algorithms. Cases where no consensus could be reached by all three senior MSK-trained observers (K.S., E.E.V. and G.A.K.) were considered as the most complicated and were used for further benchmarking of the developed machine learning method.

### 2.3. Radiomics Analysis and Machine Learning

Femoral heads and necks were manually segmented by a radiology resident with 10 years of experience in hip MRI research, with 3D Slicer (v 4.11 for Windows, https://slicer.org, date last accessed: 15 September 2021). In order to achieve gray level harmonization across STIR images from various scanners, histogram normalization and a fixed bin width were used according to the recommendations of PyRadiomics for MRI-based feature extraction (https://pyradiomics.readthedocs.io, date last accessed: 15 September 2021). Voxel spacing standardization was achieved by resampling to a voxel size of 1 × 1 × 1 mm and 849 radiomics features were extracted from the defined ROIs including original, wavelet and Laplacian of Gaussian filtered values. Radiomics features were scaled ($RF_{scaled}=\frac{RF-\mu o_{RF}}{SD_{RF}}$, *μ*: mean, *SD_RF_*: standard deviation), multicollinearities were reduced by removing highly correlated features (Pearson correlation > 0.7) and feature selection was performed with the used of the Boruta feature selection algorithm with a cutoff set at *p* < 0.01, to enable more robust machine learning model development. Boruta is a random forest-based feature selection method which has been shown to perform better in high-dimensional datasets than alternative common algorithms [22,23]. Boruta selects features useful for discriminating between the two conditions, eliminating all irrelevant features which could lead to model overfitting as previously described [24,25].

**Figure 2:**
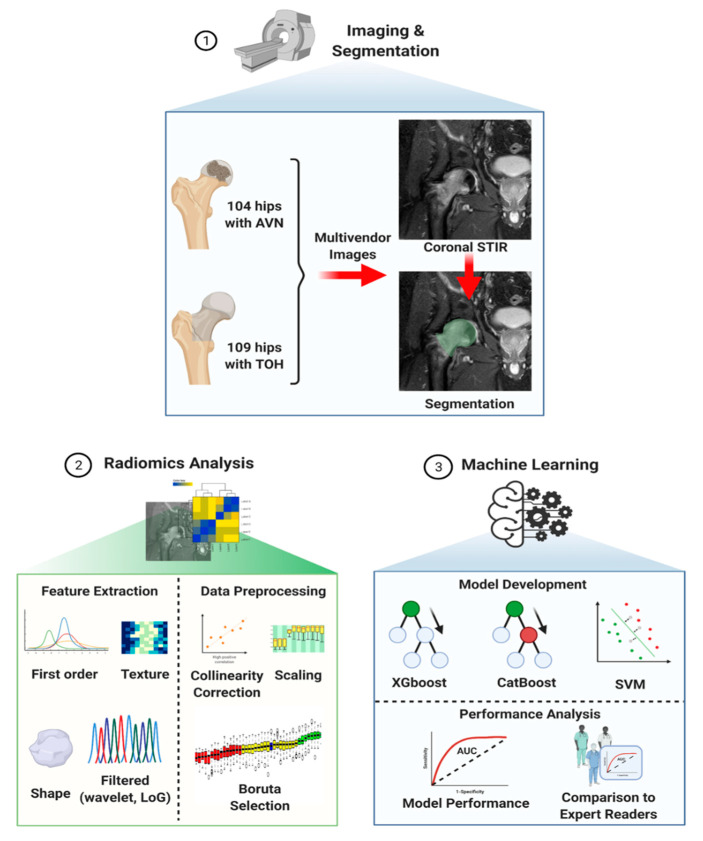
Computational pipeline for radiomics analysis and machine learning model development. The process starts with image acquisition and segmentation of the femoral head and neck (1) followed by radiomics analysis (2) consisting of feature extraction and data preprocessing in preparation for subsequent model development (3). Three machine learning algorithms (XGboost, CatBoost and SVM) were trained and validated with multivendor data and their performance was compared to that of expert readers. TOH: Transient Osteoporosis of the Hip; AVN: Avascular Necrosis; STIR: Short Tau Inversion Recovery; LoG: Laplacian of Gaussian; SVM: Support Vector Machine (created with BioRender.com, date last accessed: 15 September 2021).

The resulting curated radiomics dataset was used to build three machine learning classifiers to discriminate between TOH and AVN. The study was performed according to the Checklist for Artificial Intelligence in Medical Imaging (CLAIM) guidelines [26]. All classifiers were built with the use of the R programming language (v. 4.03, https://www.R-project.org/, date last accessed: 15 September 2021) by training two advanced gradient boosting algorithms, XGboost and CatBoost, as well as a support vector machine (SVM) model, as implemented in the packages “xgboost”, “catboost” and “e1071”, respectively. The dataset of 213 images (109 hips with TOH and 104 with AVN) was split for training and testing with a ratio of 70:30 (training:testing dataset) (Figure 1). Machine learning classifiers were developed with 10-fold cross-validation in the training dataset and hyperparameter tuning was performed with the use of random search. The testing dataset served as an external validation set, since the MRI examinations were collected from multiple scanners at different centers, ensuring that our models are not center specific. The pipeline for radiomics and machine learning analysis is described in Figure 2.

Extreme gradient boosting (XGboost) is considered the most successful algorithm for the classification of tabular data such as radiomics. XGboost is a tree-based method which builds an ensemble of classification trees. At each training step, a new random tree is created and a model is added to reduce the error of the already present trees. Stochastic gradient descent is used to minimize the loss when adding each new tree. XGboost is considered the most successful machine learning algorithm, winning the most machine learning competitions using tabular data. It offers faster execution speed and optimal accuracy to other algorithms [27,28]. Most importantly, randomization techniques and regularization are implemented in XGboost to avoid overfitting, rendering XGboost extremely lucrative in studies with small sample sizes. In addition, XGboost scales well with computer resources, adapting to the available hardware. For all these reasons, XGboost was our first choice combining all the desirable advantages required in further clinical or commercial distribution of our algorithm [28,29]. CatBoost is a newer variant of gradient boosting algorithms, which has the ability to handle both categorical and numerical data equally well and is thought to achieve optimal execution speeds, maximum accuracy and minimal overfitting [30]. Therefore, CatBoost was our second choice after XGboost since it is one of the most advanced gradient boosting algorithms with clear advantages in the case of clinical or commercial distribution of our algorithm. Support vector machine (SVM) is one of the traditional but still relevant supervised machine learning methods which finds the optimal separating margin (hyperplane) between each pair of clinical classes. SVM performs well in a wide range of classification problems [31]. Even though XGboost is known to outperform SVM in most cases of radiomics data, SVM has been widely used in radiomics studies, achieving acceptable results [32,33],. Therefore, comparison of our results with the results of SVM can offer a significant comparison to other published methodologies and traditional machine learning methods.

### 2.4. Statistical Analysis

Descriptive statistics were used to analyze patient demographics, presented as frequencies and mean ± standard deviation (SD). Sensitivity, specificity, positive predictive value (PPV) and negative predictive value (NPV) were calculated for each classifier and expert reader for the detection of AVN against TOH. Receiver operating characteristic (ROC) curves were constructed with the use of the pROC R package and classifier performance was assessed with the respective area under the curve (AUC) and 95% confidence intervals for the AUC calculated by bootstrapping. Expert reader performance (AUC) was compared to the best performing classifier with the use of DeLong’s method [34] and the respective ROC curves were plotted together. Statistical analysis was performed with the use of R (v. 4.03, https://www.R-project.org/, date last accessed: 15 September 2021) and the non-parametric Mann–Whitney U test was used to compare the ages of patients between the two groups. Significance was defined with a *p*-value lower than a = 0.05.

## 3. Results

### 3.1. Patient Demographics

The mean age of patients with AVN was 43.74 ± 14.77 years, which did not significantly differ compared to the mean age of patients with TOH, which was found to be 45.77 ± 10.3 years (*p* = 0.464). A total of 94 right and 119 left hips of 113 male and 61 female patients were included in the study (Table 1). 

### 3.2. Radiomics Analysis and Machine Learning Model Development

**Figure 3:**
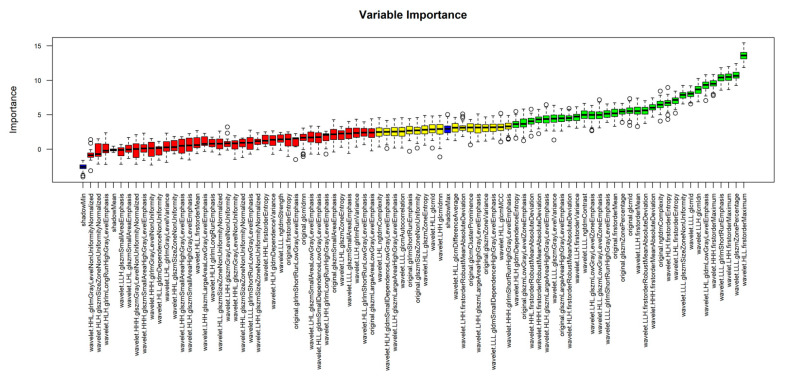
Identification of important features with the use of Boruta feature selection. Following collinearity correction and scaling, Boruta was applied as an artificial intelligence algorithm to select relevant features for unbiased development of machine learning classifiers. The Z-score boxplot presents rejected (red), tentative (yellow) and accepted (green) features. *p* < 0.01 was used as a cutoff for the selection of accepted features. Blue boxes represent Z-scores of shadow features acting as internal controls for the selection of important variables. Subsequent machine learning was performed using accepted (green) features.

**Figure 4:**
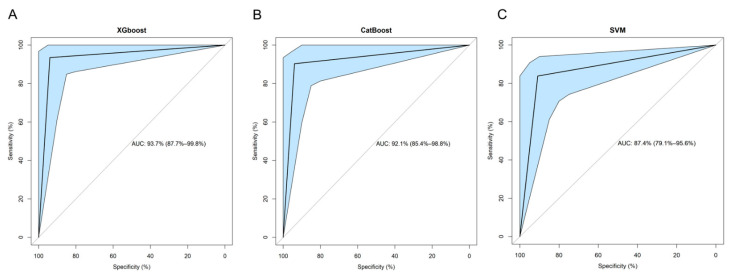
Receiver operating characteristic (ROC) curves of machine learning models. XGboost (**A**), CatBoost (**B**) and support vector machine (SVM) (**C**). Light blue areas represent the respective 95% confidence intervals calculated with bootstrapping. AUC: Area Under the Curve.

**Figure 5:**
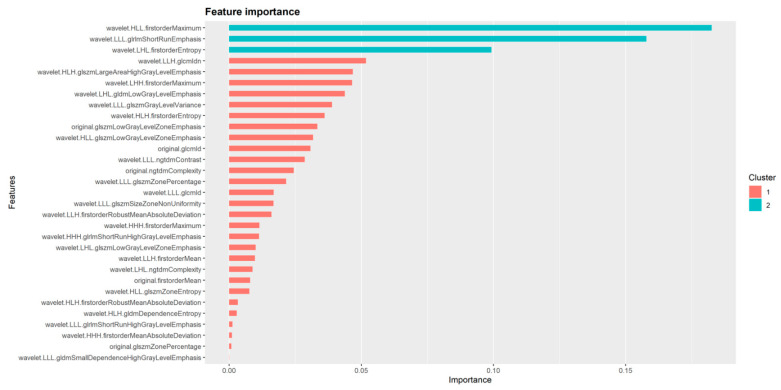
Radiomics features identified as important for the performance of XGboost. Important features belong to two clusters based on their degree of importance. Cluster 2 contains three features which represent the most important determinants of XGboost performance in differentiating between TOH and AVN.

Following data scaling and collinearity correction, Boruta was used to extract a subset of 38 radiomics features from the initial 849 feature dataset, consisting of 31 wavelet and seven original features. This set of 38 features was used for subsequent machine learning model development (Figure 3). XGboost achieved the best performance in discriminating between AVN and TOH with an AUC of 93.7% (95% CI from 87.7 to 99.8%), whereas CatBoost achieved slightly lower performance with an AUC of 92.1% (95% CI from 85.4 to 98.8%) and SVM achieved the lowest AUC of 87.4% (95% CI from 79.1 to 95.6%) (Figure 4 and Table 2). Given the superior performance of XGboost, it was utilized to identify radiomics features that play an important role in discriminating between the two conditions. Thirty one out of 38 features were found to contribute to model performance. However, the wavelet filtered maximum, short-run emphasis and entropy were found to be the three features (cluster 2 in Figure 5) with the highest importance contributing to the differentiation between the two conditions. The majority of important features (26/31) used by XGboost to accurately classify MR images were derived from wavelet decompositions of the original images (Figure 5). Performance of all three algorithms in the training set reached an AUC of 100%. The fact that the independent test set had similar performance, the use of early stopping in XGboost training and the fact that log-loss in the train and validation set continued to decline until the final iteration of the models ensured that our models did not overfit.

### 3.3. Comparison of Machine Learning to Radiologists

**Figure 6:**
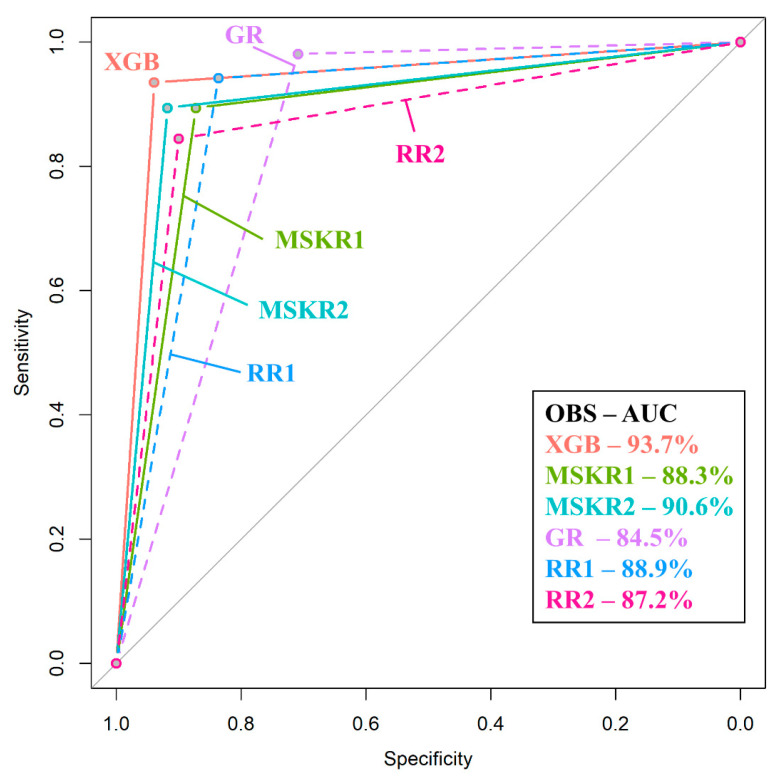
Comparison between receiver operating characteristic (ROC) curves of XGboost and expert readers. ROC curves of XGboost and musculoskeletal radiologists are plotted as solid lines whereas the ROC curves of residents and the general radiologist are plotted as dashed lines. XGboost (pink line) is shown to have the best performance, which was significantly higher than the performance of a general radiologist (GR—purple line). XGB: XGboost; MSKR: Musculoskeletal Radiologist; GR: General Radiologist; RR: Radiology Resident; OBS: Observer; AUC: Area Under the Curve.

In order to appreciate the value of the proposed method, the best performing algorithm (XGboost) was compared to expert readers. Given the difficulty in differentiating between the two entities, radiologists at various levels of training were selected to participate in the study. The highest performance was achieved by one of the two MSK radiologists who achieved an AUC of 90.6% (95% CI from 86.7% to 94.5%, *p* < 0.001) with a sensitivity of 89.42% and a specificity of 91.82%, whereas the second MSK radiologist achieved an AUC of 88.3% (95% CI from 84% to 92.7%, *p* < 0.001). Radiology residents undergoing subspecialty training in MSK radiology performed equally to fellowship-trained MSK radiologists (AUC of 88.9% and 87.2% for the 4th and the 5th year resident, respectively). Interestingly enough, MSK-oriented residents achieved a sensitivity superior to XGboost but with significantly lower specificity (70.91% and 83.64% specificity for each of the residents, respectively). The general radiologist achieved an AUC of 84.5% (95% CI from 80% to 89%, *p* < 0.001), which was significantly lower than the performance of XGboost (*p* = 0.017) which performed on average 9.2% better. The performance of all other readers was slightly lower than the model without reaching significance (*p* > 0.05) (Figure 6 and Table 3). The performance of XGboost was also evaluated against a series of the most complicated cases of our dataset (where no consensus agreement could be made by the three senior MSK-trained observers), where it achieved an AUC of 91.7% (95% CI 75.3–100%) (examples shown in Figure S1).

## 4. Discussion

Differentiation between TOH and AVN represents a challenging task for radiologists because of similarities in their imaging appearance, including the presence of BME, subchondral fractures and femoral head collapse in advanced disease. Accurate diagnosis requires significant expertise and evaluation of clinical data including pain characteristics and risk factors. Herein, discrimination between the two entities has been achieved with the use of radiomics and machine learning. Most importantly, multivendor imaging data were used to develop an XGboost classifier which performed significantly better than a general radiologist and equally to MSK radiologists and MSK-oriented radiology residents.

Several studies have attempted to discriminate between TOH and AVN based on their imaging appearance. Klontzas et al. presented a series of 155 patients with TOH with a follow-up between 1 and 10 years, demonstrating that microtrabecular insufficiency subchondral fractures can be present in approximately half of these patients, which have a completely different appearance compared to necrotic lesions of AVN [5]. They also showed that another unique feature of TOH is the “sparing” sign which represents sparing of the medial bone marrow of the femoral head by BME in 87.7% of TOH cases [5]. On the other hand, AVN is complicated with BME at later stages of the disease resulting from articular collapse and neither the “band-like” nor the “crescent” sign resemble the subchondral insufficiency fractures of TOH [5,9,21]. This is corroborated by data showing that subchondral fractures of TOH never progress to AVN [5,9]. Despite the accumulating data defining the imaging differences between the two diseases, MSK radiologists still face difficulties in diagnosis especially in the absence of accompanying clinical data and confusion is still evident in recent literature [11,12]. Attempts have been made to differentiate between the two entities using dynamic contrast-enhanced MRI, but more patients are needed to account for various AVN stages [35]. Towards this end, radiomics and machine learning have achieved a diagnostic performance with an AUC close to 95%, equal to MSK radiologists and significantly better than a general radiologist, without the incorporation of any clinical data by both of the radiologists and the algorithm. The performance of our algorithm was found to be excellent even when it was specifically assessed against the most complicated of cases in our dataset, indicating the diagnostic value of the algorithm in everyday clinical dilemmas. This powerful machine learning strategy can assist hip MRI reporting by experienced and inexperienced radiologists, protecting TOH patients from unnecessary surgery and ensuring prompt management of patients with AVN, in order to prevent articular collapse and total hip replacement. In everyday clinical reality, when evaluating MRI examinations with atraumatic bone marrow edema, no other entity can complicate the differential diagnosis by imitating either AVN or TOH. Therefore, differentiation between the two entities presents a real-life diagnostic challenge and cases where radiologists are asked to decide between the two is a common occurrence in centers specialized in bone marrow imaging.

The majority of features found to be important for the discrimination between the two diseases were wavelet transformations of original radiomics features. This can be potentially explained by the type of imaging characteristics used by the human eye for the traditional diagnosis of TOH and AVN. Traditional MRI diagnosis is based on the presence of subchondral band-like serpiginous changes, the “crescent” sign, BME and its pattern and low signal intensity irregular thin lines inside BME, located deep in the femoral head [4,21]. All these characteristics are largely composed of edges and irregular lines which are well known to be enhanced when images undergo wavelet transformation at specific scales [36,37]. At the same time, although the images were normalized prior to machine learning modeling, inconsistencies in the noise profile and the image contrast emanating from the heterogeneous imaging conditions and protocols of the multicentric dataset used are still expected to exist. Wavelet decomposition addresses this problem by combining high and low pass filters to separately examine different frequency ranges across the dataset. It could be then postulated that the high performance of the presented models is strongly dependent on wavelet decomposition by highlighting edge information and suppressing sources of variability in the original MR images.

Our study has certain strengths and limitations. Given the low prevalence of both entities and especially TOH, the large number of hips examined in this study is an important strength. In addition, the use of MR images from multiple vendors significantly increases the value of our study, enhancing the generalizability of our results since our models have been trained in handling data acquired at multiple sites. Another strength of our study is the comparison of machine learning to radiologists which provides an objective estimate of the importance of this work. One of the limitations of our study is its retrospective nature. However, retrospective analysis is mandated by the low prevalence of TOH and AVN along with the large number of images required for machine learning. Manual segmentation could also be a limitation for our method. However, in our case, segmentation involved selecting the outline/cortex of well-defined bone structures (femoral head and neck) without involving subjective segmentation of single or ill-defined lesions that could potentially introduce bias. Finally, the sole use of STIR images in this study could potentially be considered a limitation of our study. Nonetheless, in everyday radiological practice, fluid-sensitive sequences (e.g., STIR, proton-density weighted with fat suppression) suffice for the diagnosis of both AVN and TOH, since they are sensitive for the identification of bone marrow edema patterns characteristic to both diseases.

## 5. Conclusions

In conclusion, this study presents a radiomics-based machine learning method for differentiating between TOH and AVN. Machine learning achieved a performance similar to MSK radiologists and significantly higher compared to general radiologists. The pipeline presented in this manuscript can be used to aid the diagnostic process, protecting TOH patients from unnecessary surgery as a result of misdiagnosis.

## Figures and Tables

**Table 1:** Patient demographics.

	Total	AVN Hips	TOH Hips
Number of hips	213	104	109
Age	44.76 ± 12.53 years	43.74 ± 14.77 years	45.77 ± 10.3 years
Side	94R–119L	56L–48R	63L–46R
Sex *	61F–113M	38F–29M	23F–84M

*: number of patients; AVN: avascular necrosis; TOH: transient osteoporosis of the hip; F: female; M: male; R: right; L: left.

**Table 2:** Performance of the three machine learning algorithms.

Performance Measure	XGB	CB	SVM
AUC (95% CI)	93.74% (87.7–99.8%)	92.1% (85.4–98.8%)	87.4% (79.1–95.6%)
Sensitivity	93.55%	90.32%	83.87%
Specificity	93.94%	93.94%	90.91%
PPV	93.55%	93.33%	89.66%
NPV	93.94%	91.18%	85.71%
*p*-value	<0.001		

AUC: Area Under the Curve; CI: Confidence interval; XGB:XGboost; CB: CatBoost; SVM: Support Vector Machines; PPV: positive predictive value; NPV: negative predictive value.

**Table 3:** Comparison of XGboost to expert readers.

Performance Measure	XGB	MSKR1	MSKR2	GR	RR1	RR2
AUC (95% CI)	93.74% (87.7–99.8%)	90.6% (86.7–94.5%)	88.3% (84–92.7%)	84.5% (80–89%)	88.9% (84.8–93.1%)	87.2% (82.7–91.7%)
Sensitivity	93.55%	89.42%	89.42%	98.08%	94.23%	84.47%
Specificity	93.94%	91.82%	87.27%	70.91%	83.64%	90%
PPV	93.55%	91.18%	86.92%	76.12%	84.48%	88.78%
NPV	93.94%	90.18%	89.72%	97.50%	93.88%	86.09%
	*p*-value *	0.39	0.15	0.017 **	0.19	0.08

AUC: Area Under the Curve; CI: Confidence interval; CR:Consultant radiologist; RR: radiology resident; PPV: positive predictive value; NPV: negative predictive value; *: *p*-value of the comparison of each reader to XGB; **: statistically significant value.

## Data Availability

Data not contained in the manuscript will be available from the corresponding author by reasonable request.

## Supplementary Materials

The following are available online at https://www.mdpi.com/article/10.3390/diagnostics11091686/s1, Figure S1: Examples of cases where differential diagnosis between avascular necrosis (AVN) and transient osteoporosis of the hip (TOH) can be complicated.
