# Supplementary figures and images for: Radiomics and Machine Learning Can Differentiate Transient Osteoporosis from Avascular Necrosis of the Hip
Source: Diagnostics (Basel). 2021 Sep 15;11(9):1686. doi: 10.3390/diagnostics11091686 (PMC8468167; doi:10.3390/diagnostics11091686)

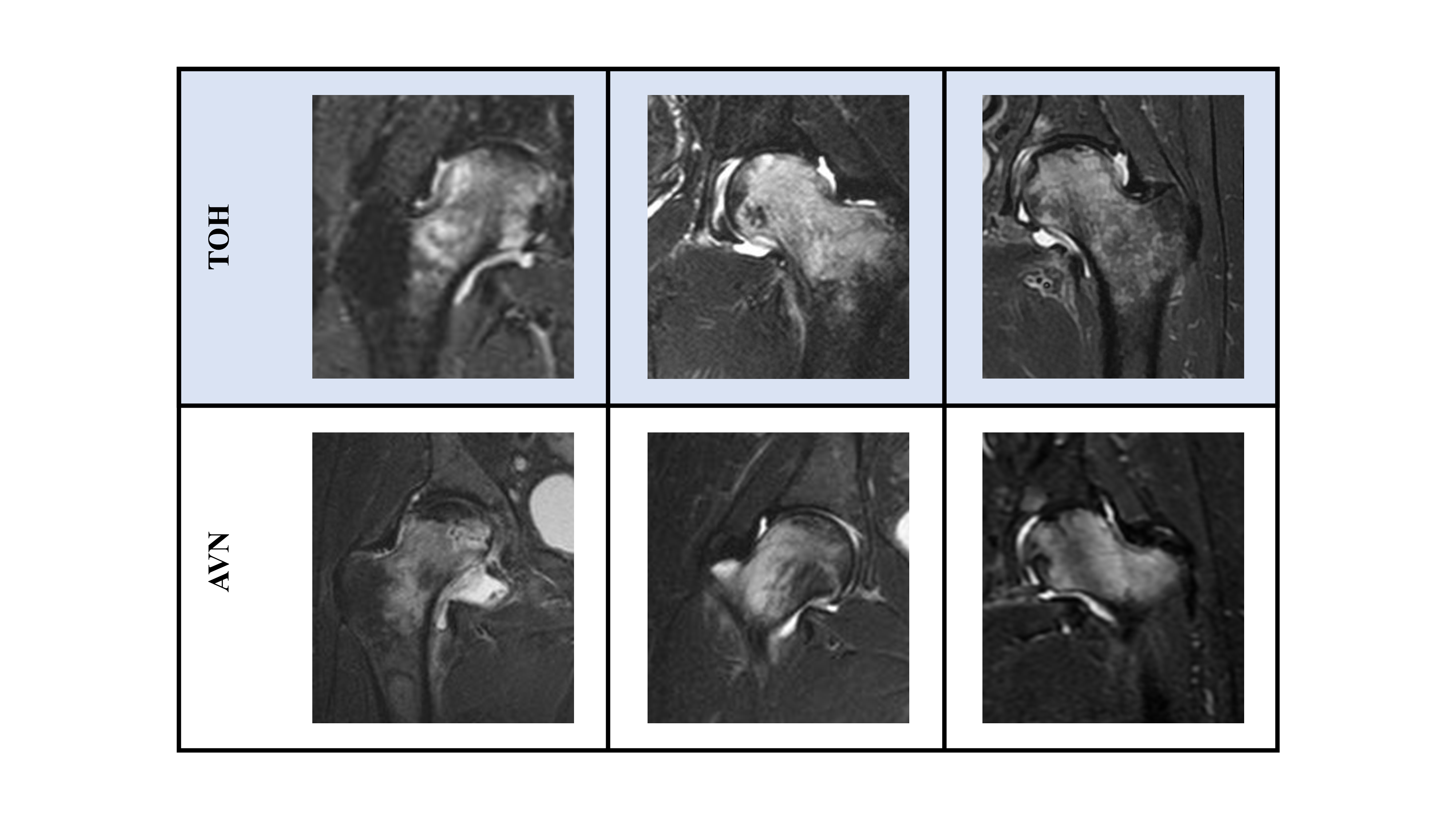

Supplement: Supplementary file 1 [file diagnostics-11-01686-s001.zip › diagnostics-1361935-supplementary.png]
